# Nerve-Storms

**Published:** 1877-01

**Authors:** P. H. Hayes


### NERVE-STORMS--THEIR TREATMENT.

P. H. HAYES, M. D.

The first and best element of all treatment is *prevention.* To see the storm *a-brewing* and hasten to defeat it is the best of all medical skill, as it is also the highest benevolence to him who in practice knows something more than blue pills, iodine and mustard plasters.

I was told a shocking story a few days ago by a very intelligent young lady, a teacher in one of our public schools, who says that a nervous child had a habit of clinging to her whenever she could get an opportunity. Interesting herself more and more in this child, she observed a habit of twitching, and on speaking to the pale little girl about it, she said that on the previous Sunday she handed her plate for food, and her arm jerked so she let her plate fall, and her mother whipped her for it. This thoroughly roused the teacher, she took the child home and told the mother the child needed a physician and not the rod.

Will you believe it? Dr. A. McLane Hamilton, declares that from his investigations, one-fifth of all the smaller children in our public schools twitch their hands or faces on one side of the body, and that about one-sixth part of all are subject to headaches. If such children as these are allowed to attend school where pressure is constantly brought to bear to make them keep up with their classes, what wonder that we have so much hysteria, epilepsy, neuralgia, sick-headache and spasmodic dysmenorrhœa to treat in after life.

We know that in nearly all these cases there is a hereditary tendency to these diseases, yet this makes the pressure of our school system all the more dangerous, for it is right here that the latent tendency is developed into actual disease, which under other circumstances might never have happened. We ought surely to have a bureau of inspection and inquiry to ascertain what children are and what are not able to go to school. I have in mind this moment a young man, a hopeless epileptic, whose slight attacks—the *petit mal*—began in the school-room. I constantly notice in my own children an increased sensitiveness to all mental and bodily influences as soon as they begin school. If they come down in the morning irritable and fault-finding, I know they have been studying the previous evening, they have set up late and have not had *sufficient sleep.* Take care about this irritability of temper, it is always disease, the sign of something wrong in the nervous system or digestive organs, or both, and when it mounts to passion it is a true nerve-storm, and not far off from chorea, epilepsy or melancholy.

Is it not amazing how near to disease are many perfectly normal and healthy actions. Sneezing is a true nerve-storm of an eminently explosive kind and truly normal and healthy, yet in asthma and in some other conditions of disease persons have been known to sneeze thirty or forty times in succession, and until they were exhausted by the act. This condition of things is as truly a nervous disease—a *neurosis*—as the spasms of St. Vitus dance. Indeed, those who *dance* attendance on their noses at these times would cheerfully admit the dance, but would not name it after any saint I

trow. The natural appetite for food is capable of becoming so preverted as to amount to a real nervous disease, as when hysterical girls rejecting wholesome food, crave only unwholesomeness, or have recourse to chalk, charcoal, &c. No act is more normal and healthy than *laughing*, yet in certain persons it is a dangerous amusement. A healthy nerve-storm quickly develops a diseased one. Thus Dr. Salter declares that laughing will develop asthma, and that it does so in a great many cases, and he says he has known numerous instances of *hay fever* in which the sufferers dared not laugh for fear of bringing on the asthma, a thing they had been taught by experience was no laughing matter. *Coughing* and *sneezing* will do the same when violent and long continued. Dr. Laycock cites cases of minor epilepsy brought on by laughing. Again a fit of laughter may be brought on by tickling the soles of the feet, and in those pre-disposed to epilepsy an attack may be induced in the same manner. Nay, in the celebrated case found in Van Sweiten, there was no pre-disposition whatever. I give the case in his own words:

"I have seen," he says, "a very healthy girl of ten years old, the offspring of vigorous parents, who never had epilepsy, rendered epileptic for many years, and seized with her first paroxysm while some girls in play tickled the soles of her feet, others holding her down so that she could not escape the intolerable sensation." A more striking instance still is recorded by Dr. Reynolds in his excellent work on epilepsy. A boy of fifteen had his first attack from having his feet tickled while he was asleep. The tickling did not awaken him nor make him laugh in his sleep, but excited instant epilepsy.

Following up the idea of preventing nerve-storms of a morbid kind, I must notice the curious fact that they are all *catching*. How often have the best of us found ourselves yawning because some one else near us did so, and when once begun a little epidemic of yawning will go the rounds over and over again of a room full.

Just so we have heard a whole church full cough almost simultaneously, or *ahem* to clear their throats because some *one* or two who had bronchitis and must do it, had set the example. These are harmless and healthy acts, but when a child becomes epileptic from seeing a fit, or women have hysteria from witnessing it in others, which has repeatedly happened, then it becomes a serious matter. Dr. Bright mentions being called to a case of chorea in a young girl where neither she nor her friends could give any reason whatever for her having the disease, but on inquiring if she knew any one with her complaint it was found that a school-mate had the disease. The author of this article knows a case where stammering was similarly developed and could never afterward be perfectly cured.

In our last article we tried to show how naturally a large number of nervous diseases of the spasmodic and epileptiform type were grouped together under the general title of *nerve-storms*, and we enumerated as belonging to this group epilepsy, asthma, angina pectoris, chorea, hysteria, neuralgia and megrim in its various forms of hemicrania, bilious headache, brow ague, sick giddiness and blind headache. We will suppose any one of these diseases to have become established in the system, we shall find that it is kept up by debility, by habit, and by mental conditions, and we must meet and conquer all these several conditions of disease by a thorough system of hygienic and medicinal means.

As to the first, debility, we all know that *life* and *death* are opposed to each other, but we have not stopped to think that *life* and *disease* are just as truly opposed to each other and for the same reason. Disease is a warfare against life and on the death side, Life is a warfare against disease and on the life side. Now whatever renews the powers and actions of life by strengthening them, whatever treatment will add continually *more life* to the body, will cause chronic diseases to fade out and disappear as the advancing light of the morning dispels the darkness of the night. If asked what will cure any case of chronic disease, we answer *more life*.

Let us notice right here the power of habit over the organic actions of the body. We see this in health, the return of hunger at stated and regular times, of the desire for sleep, of the motions of the bowels, yet these habits can be greatly changed to suit altered circumstances. Thus the policeman whose beat is around yonder street corner, has waked from his day-time sleep at sundown, and then takes his breakfast and will take his dinner at midnight. A man who gives way to anger, to avarice, to sexual passion, will soon find he is developing powers within himself by habit which turn furious tyrants on slighter and slighter provocations. It can hardly be doubted that many cases of epilepsy are kept up in great part by habit. Old Dr. Fountain told me that he had broken up the attacks of epilepsy in these cases by the use of tartar emetic in an emetic dose the instant the warning made its appearance. Prof. Hammond says let the epileptic patient who has any warning, carry with him a vial containing just five drops of the nitrite of amyl, and inhale instantly on the warning, and it will prevent the fit every time. As to St. Vitus dance and whooping cough, it is probable that habit will perpetuate them long after the disease has really disappeared. Sir John Herschel could bring on his megrim by allowing his mind to dwell on its symptoms. M. Piorry, an eminent French pathologist, could bring on his megrim by fixing his sight intently. Such slight causes may induce a disease to which the system has become habituated. Esquirol declares that the habit of simulating epilepsy has actually induced the genuine disease. It must greatly tax the intelligence of the physician to judge just how far his patient's disease is a pathological habit, and then to arouse the patient's will and devise the means to break the power of habit. Dr. Salter most ingenuiously remarks:—"It seems as if it were a law of disease to continue its present type and peculiarities whatever they may be until something occurs to 'break the spell,' and then to continue the acquired change whatever that may be, until something else occurs, and so on."

As to the mental conditions which often come in to keep up paroxysmal disorders, fright, anger, dread, over-anxiety and mental over-work. Authors set down a large proportion of cases of epilepsy as caused by fright. So also anxiety of mind exhausts the nervous system and induces many attacks. John Hunter brought on a fierce attack of angina pectoris by a fit of anger, which cost him his life. Fear has often brought on hysteria, spasmodic colic and the asthma. Dr. Salter mentions the case of a boy who would say to his father, "don't scold me or I shall have the asthma," and sure enough it would certainly come whenever he was corrected. In a patient of my own a very prolonged siege of megrim followed some weeks of suspense and anxiety as to her absent sons.

Now just as depressing emotions and passions excite these diseases, so the opposite emotions tend to cure. It should therefore be one of the first aims of the physician to create a new hope for his patient, give them a new inspiration, something new to think of, some new outlook in their lives.

A young woman came under my care with epileptiform neuralgia. She would be compelled to rise at night and walk her room with the agony of the pain. Her improvement was very slow until I perceiving in her a marked turn for scientific study advised her to study medicine. She entered into the study with a wonderful enthusiasm, and then everyting done for her disease did her good. Her neuralgia soon disappeared. She graduated in medicine and entered upon its practice.

Sometimes it is the digestive system that requires special care and treatment. Sometimes there is disease of the liver and poverty of the blood as a basis, and upon removing these the disease is cured. Change of climate is one of the best of all measures and will of itself cure many of these maladies. It overcomes debility, it tends to break the power of habit in inducing the disease, and it makes up a new and healthy emotional life. The physician will find in salt-bathing and electricity resources of great power when wisely adapted to the case. Finally we must not omit to mention that as overtaxing the eyes is a very common cause of neuralgia, and especially of the various forms of megrim, this must not be forgotten in our treatment.

### CLAIRVOYANCE.

Those who believe in clairvoyance and clairaudience—the ability to see without the eyes and hear without the ears—are mortally puzzled by cases like this of the professional clairvoyant, Mme. Florence, who, losing her child by abduction, straightway loses also faith in her mystic power, and instead of putting her mental optics to the test, cries aloud and hands the case over to the police. This is discouraging. It was such a chance for proselytism. If Mme. Florence had ignored the police, or had summoned them merely to see the better work of the invisible detective who runs without legs, like the traveler on the enchanted carpet, and if she then had put a wet towel around her head and tracked the false and fugacious Minnie Wood to her lair, she would have made a sensation and multiplied converts to her recondite profession. But when she closes those sleepless eyes of the spirit and wails helplessly for the police, the distressed lady finds her child but loses her reputation.

A NEW TEST-PAPER.—A new test-paper, invented by Waller, is, says the *Chemical News*, prepared by soaking strips of white paper in a solution of coralline. It is exceedingly sensitive to the action of alkalies; very dilute alkaline liquids instantly turn it a beautiful red color. Acids turn it yellow; but, as the reaction is less striking, Dr. Waller proposes to use it only for alkalies, as a substitute for red litmus.—*The Medical Press and Circular.*

